# The effect of age on heart rate variability indices during and following high-intensity continuous exercise in masters and young cyclists

**DOI:** 10.1007/s00421-024-05588-y

**Published:** 2024-08-30

**Authors:** Nattai R. Borges, Peter R. Reaburn, Scott Michael, Thomas M. Doering

**Affiliations:** 1 Faculty of Medicine and Health, School of Health Sciences, University of New South Wales, Sydney, NSW 2052 Australia; 2 College of Health, Medicine and Wellbeing, School of Biomedical Sciences and Pharmacy, University of Newcastle, Callaghan, Australia; 3 Bond Institute of Health and Sport, Bond University, Gold Coast, Australia; 4 Institute for Health and Sport, Victoria University, Victoria, Australia; 5 School of Health, Medical and Applied Sciences, Central Queensland University, Rockhampton, Australia

**Keywords:** Older athlete, Parasympathetic reactivation, Cycle, Recovery

## Abstract

This study compared heart rate variability (HRV) parameters of cardiovascular autonomic regulation between well-trained masters and young cyclists at rest, during and following a continuous cycle (CTS) protocol. Ten masters (age = 56 ± 5 years) and eight young (age = 26 ± 3 years) cyclists completed a 100 min experimental protocol consisting of a 60 min CTS cycling bout at 95% of Ventilatory Threshold 2 followed by 40 min of supine recovery. Beat-to-beat heart rate was measured continuously, and HRV parameters analysed at standardised 5 min intervals during rest, exercise and recovery. The root mean square of the successive differences (RMSSD), low-frequency power and high-frequency power parameters were corrected by division of the R-R interval (time domain), or R-R interval squared (frequency domain). Further, the RMSSD and RMSSD:RR for successive 60-s R-R intervals at the onset (0–10 min) and offset (60–70 min) of CTS exercise were analysed over 10-min periods. The natural logarithm (Ln) of skewed parameters was taken for analysis. Significant interaction effects (*P* < 0.05) for 5 min segments were found for (LnRMSSD, LnRMSSD:RR, LnHF and LnHF:RR^2^. Masters cyclists demonstrated lower values of parasympathetic activity at rest and during recovery (15–20 min and 35–40 min) compared to younger cyclists. However, similar HRV responses were observed during exercise, including throughout the acute onset and offset periods (*P* > 0.05). This data shows that cardiac autonomic regulation during, or immediately following CTS exercise may not be influenced by age, but masters athletes may exhibit a lower baseline for parasympathetic activity.

## Introduction

The ageing process is generally understood to coincide with changes in autonomic regulation, in particular a reduction in parasympathetic activity (Abhishekh et al. 2013). Changes in autonomic regulation have been suggested to be a strong predictor of all-cause mortality (Messinger-Rapport et al. 2003) and coronary artery disease (Lipinski et al. 2004), as well suggesting a possible delay in acute performance recovery following exercise athletes (Plews et al. 2012). Recently, there has been increased interest in diverse cardiac measures of autonomic regulation due to the development of non-invasive measurement techniques such as heart rate variability (HRV) (Michael et al. 2017a). The measurement of HRV involves the quantification of the beat-to-beat fluctuations in heart rate, termed the R-R intervals. With the advent of commercially available heart rate monitors capable of calculating R-R intervals, the analysis of HRV to quantify autonomic regulation during exercise in diverse populations has become more common.

Research has demonstrated that short-term resting and 24-h HRV parameters of parasympathetic activity are influenced by age (Zhang 2007; Almeida-Santos et al. 2016) and physical fitness (Kiss et al. 2016). Additionally, HRV parameters at the onset (D’Agosto et al. 2014) and during exercise (Tulppo et al. 1998) have been shown to be impacted by physical fitness, and HRV in post-exercise recovery is impacted by exercise intensity (Michael et al. 2016, 2017b). Furthermore, amongst older adults, short-term resting and 24-h HRV have been reported to be higher in physically active compared with sedentary participants (Ueno and Moritani 2003; Soares-Miranda et al. 2014). Therefore, physical fitness and training seem to have a protective influence on HRV parameters of cardiac autonomic regulation into older age. However, it remains difficult to elucidate the potential protective influence of life-long exercise on autonomic regulation without longitudinal training studies.

Through continued participation in competitive sports and systematic training, masters athletes have historically demonstrated the ability to attenuate declines in physical (Borges et al. 2018; Fernandes et al. 2020) and physiological performance into older age (Borges et al. 2016, 2020; Lepers and Stapley 2016). Masters athletes are defined as individuals who systematically train and compete in organised forms of competitive sport specifically designed for older adults (Reaburn and Dascombe 2009). Several investigations have demonstrated that masters athletes exhibit improved indices of parasympathetic activity at rest compared with age-matched sedentary counterparts (Leti and Bricout 2013; Deus et al. 2019; Sotiriou et al. 2013; Galetta et al. 2005) and comparable parasympathetic reactivation following exercise compared to younger athletes following high-intensity interval exercise (Borges et al. 2017).

Collectively, these studies suggest that systematic long-term training may attenuate detrimental age-related changes in cardiac autonomic regulation. However, no studies have investigated the age-related HRV response during exercise in masters athletes, and further research is required to understand any age-related influence on HRV following exercise of different intensities that represent common training protocols utilised by masters athletes. Given the current popularity of monitoring HRV for recovery, a greater understanding of these responses may provide further context for the role of HRV in quantifying training stress and recovery in masters athletes. Therefore, the aim of this study was to compare HRV-derived parameters of cardiac autonomic regulation in well-trained masters and young cyclists during and following a high-intensity continuous cycle (CTS) protocol.

## Methods

### Participants

Ten masters (age: 55.6 ± 5.0 yr) and eight young (age: 25.9 ± 3.0 yr) well-trained cyclists were recruited from local cycling and triathlon clubs (Table 1). All participants were required to be free from injury and medication that may have affected their ability to perform exercise, meet a minimum VO_2_max requirement of 45 mL·kg^−1^·min^−1^, and be involved in competitive cycling or triathlon over the past two years. Prior to inclusion, participants were familiarised with the study protocol and provided written informed consent. The study was approved by an institutional Human Ethics Research Panel in accordance with the Helsinki Declaration.

**Table 1:** Mean ± standard deviation baseline data for masters and young cyclists

Group	Age (years)	Height (cm)	Body mass (kg)	VO_2max_ (mL·kg^−1^·min^−1^)	Peak power output (W)	Maximal heart rate (bpm)	Distance per week (km)
Masters (*n* = 10)	55.6 ± 5.0*	178.9 ± 8.2	81.6 ± 8.5	55.4 ± 10.4	353.2 ± 32.6	168.1 ± 13.5*	228.0 ± 69.6
Young (*n* = 8)	25.9 ± 3.0	177.8 ± 5.8	79.1 ± 4.9	62.0 ± 9.8	364.2 ± 37.0	189.0 ± 3.9	213.1 ± 128.7

^*^Significant difference between masters and young cyclists (*P* < 0.05)

### Study overview

Testing was completed over two sessions separated by at least 48 h at the Exercise Physiology Laboratory at CQUniversity. Environmental conditions for the laboratory were standardised at 22 ± 2 °C and < 70% relative humidity. The first session consisted of preliminary testing included the determination of VO_2max_ and ventilatory thresholds. The second session consisted of the experimental protocol, including a high-intensity 60 min CTS cycle bout followed by 40 min of supine recovery. Exercise testing was performed on an electromagnetically braked cycle ergometer (Velotron, Racermate; Seattle, USA). Both seat and handlebar positions were adjusted according to the preference of each participant and replicated for each testing session. Participants were asked to follow their usual dietary intake and not perform strenuous exercise during the 48 h preceding any testing. Additionally, participants were requested to not consume any caffeine on the day of testing and not to have a large meal three hours prior to coming to the laboratory.

### Preliminary testing

Preliminary testing consisted of familiarisation of testing protocols, collection of demographic data (height, mass) and a maximal graded exercise test (GXT). The GXT was preceded by a standard warm-up of 6 min at 100 Watts (W). The GXT commenced at 150 W and work rate increased 50 W every 3 min until volitional exhaustion. Expired gas was continuously analysed throughout using an indirect calorimetry system (TrueOne 2400*,* Parvo Medics, Inc.; Sandy, USA) calibrated according to the manufacturer’s instructions prior to each test. Ventilatory thresholds one and two (VT1 and VT2) were determined using ventilation equivalents as described by Lucia et al. (2000). Briefly, VT1 was defined as the first marked increase in the ratio between expired ventilation and oxygen consumption (V_E_:VO_2_) with no concomitant increase in the ratio between expired ventilation and carbon dioxide production (V_E_:VCO_2_). VT2 was determined using the criteria of an increase in both V_E_:VO_2_ and V_E_:VCO_2_. Ventilation thresholds were determined for all participants by a single researcher (NB) and confirmed by a second researcher (TD). Peak gas exchange values and peak power output (PPO) were also obtained from the GXT (Hawley and Noakes 1992), PPO was calculated using the following formula:

$${\text{PPO }} = \, W_{({\text{final}})} + \, \left( {t/180 \times 50} \right)$$

where PPO = maximal aerobic power; *W*_(final)_ = Work rate (*W*) of final completed stage; *t* = duration of the final work rate completed (*s*).

### Experimental protocol

Upon arrival at the laboratory, participants commenced 10 min of supine rest to obtain a measure of resting HRV. Participants then completed a standardised 6 min warm-up at 50 W before commencing exercise which consisted of 1 h cycling at 95% of VT2. This approximate intensity has previously been reported to be self-selected in experienced endurance athletes during cycling time trials of 30–60 min (Perrey et al. 2003; Myburgh et al. 2001). Upon completion of exercise, participants were immediately (< 5 s) transferred to a plinth adjacent to the cycle ergometer for 40 min of supine recovery. Heart rate data were continuously monitored across the entire rest, exercise and recovery protocol. All resting and recovery heart rate data were obtained with the participant laying supine in a quiet dark room in accordance with previous investigations (Seiler et al. 2007; Stuckey et al. 2012). Respiratory rates (Breath min^−1^) were monitored using the indirect calorimetry system throughout the initial rest period, and at standardised 5 min intervals during the exercise and recovery period to confirm between-group responses were similar. Respiratory rate was not controlled during the recovery period as the researchers postulated that standardising the respiratory rate may have placed participants under greater stress and influenced post-exercise recovery response (Buchheit et al. 2007).

### Heart rate variability

Beat-to-beat heart rate was recorded during sessions using Polar RS800cx monitors (Polar Electro; Kempele, Finland), which have been shown to be valid and reliable to measure R-R intervals for HRV analysis (Williams et al. 2016). Five-minute R-R interval segments were analysed at rest, during exercise at 15–20 min, 35–40 min and 55–60 min, and during recovery at 15–20 min and 35–40 min. To examine the immediate cardiovascular autonomic responses throughout the rest-exercise-rest transitions, 60-s rolling R-R interval segments were analysed at the onset of exercise (0–10 min on exercise) and offset of exercise (0–10 min of recovery). Data were sampled at 1000 Hz and downloaded using the *Polar Pro Trainer 5* software (Polar Electro; Kempele, Finland).

Data taken from the *Polar Pro Trainer 5* software were converted to a text file and analysed with *Kubios HRV Analysis Software v2.2* (Biosignal Laboratory, University of Kuopio, Finland) (Tarvainen et al. 2014). Occasional ectopic beats were examined and erratic data were identified and replaced with interpolated adjacent R-R intervals using a medium artefact correction (± 0.25 s of local mean). No data were corrected more than 5% to ensure that data were not over smoothed. The mean R-R interval (ms) and the root mean square of the successive differences [RMSSD (ms)] for each 5-min segment were obtained from time domain analysis. Power spectrum analysis of the 5-min segments was performed using Fast-Fourier Transform (FFT) after data were de-trended and resampled at 5 Hz to calculate the low frequency [LF (ms^2^); 0.04–0.15 Hz] and high frequency [HF (ms^2^); 0.15–1.0 Hz] spectral power. Normalised LF power (LFnu = LF/[LF + HF] and normalised HF power (HFnu = HF/[LF + HF)]) were also derived for each 5-min segment in the frequency domain. Low-frequency measures have been suggested to be potentially reflective of baroreflex function (Rahman et al. 2011), whilst RMSSD and HF parameters are generally accepted measures of parasympathetic activity (Task 1996).

In addition to traditional HRV parameters, the RMSSD, LF and HF variables were corrected for the average heart rate response to reduce mathematic biases that may have occurred due to age-related differences in average heart rate (Billman 2013; Sacha 2013). Specifically, the RMSSD was corrected via division by the average R-R interval (RMSSD:RR) whilst the LF and HF were corrected via division by the average R-R interval squared (LnLF:RR^2^ and LnHF:RR^2^) (Billman 2013).

During the first ten minutes of exercise (onset) and recovery (offset), both the RMSSD and RMSSD:RR were analysed over 60-s non-overlapping segments to compare a time-varying vagal parameter of parasympathetic activity (Task 1996). This method has been previously used primarily in offset data (Goldberger et al. 2006; Kaikkonen et al. 2007; Martinmäki and Rusko 2008) and has been suggested to overcome potential methodological limitations of performing conventional HRV analysis during dynamic cardiovascular conditions, such as immediately after exercise (Task Force Electrophysiology 1996).

### Statistical analyses

All data are presented as mean ± standard deviations (SD) unless stated otherwise. The distribution of all data was tested for normality with the Shapiro–Wilk test. When data were skewed, they were transformed using the natural logarithm (Ln). The natural logarithm was taken for RMSSD, RMSSD:RR, LF, LF:RR^2^, HF, and HF:RR^2^ parameters using the original values for RMSSD, LF and HF and the finalised corrected values for RMSSD:RR, LF:RR^2^, and HF:RR^2^. Independent *t* tests were used to compare between-group differences for demographic data. For each HRV measure, a two-way repeated measures ANOVA was utilised to examine the interaction effects between age and protocol timepoints (i.e. group*time interactions). When statistical significance was identified, Fisher’s least significant difference post hoc analyses were used to further investigate between-group differences. All data were assessed using Mauchly’s test for sphericity and whenever a test was violated the Greenhouse–Geisser Adjustment was used. All statistical analyses were conducted using Statistica software package (StatSoft. Inc., Tulsa, USA). Statistical significance was accepted at *P* < 0.05.

## Results

No significant differences between age groups were found for demographic data (*P* > 0.05), except for age (*P* < 0.001) and maximal heart rate (*P* < 0.001) (Table 1). Additionally, there were no significant differences in the prescribed power output for the CTS bout (Masters = 236.0 ± 26.7 W; Young = 231.3 ± 18.9 W; *P* > 0.05).

Table 2 shows time domain and power spectrum HRV parameters, as well as the respiratory rate comparisons at rest, during the CTS bout and throughout recovery for masters and young cyclists. Significant group*time interaction effects were reported for LnRMSSD (*P* = 0.027; Eta = 0.20), LnRMSSD:RR (*P* = 0.022; Eta = 0.21), LnHF (*P* = 0.042; Eta = 0.18) and LnHF:RR^2^ (*P* = 0.039; Eta = 0.18). Post hoc analysis shows masters cyclists had significantly lower values for LnRMSSD, LnRMSSD:RR, LnHF, and LnHF:RR^2^ at rest (*P* < 0.01) and 35–40 min of recovery (*P* < 0.05). In addition, the masters cyclists also demonstrated lower values for LnRMSSD:RR and LnHF:RR^2^ at 15–20 min of recovery (*P* < 0.05).

**Table 2:** Mean ± standard deviation of the resting, exercise and recovery heart rate variability and respiratory rate for masters and young cyclists

	Rest			Exercise									Recovery				
				15–20 Minutes			35–40 Minutes			55–60 Minutes			15–20 Minutes			35–40 Minutes	
	Masters	Young	Masters		Young	Masters		Young	Masters		Young	Masters		Young	Masters		Young
RR (ms)	1013.41 ± 158.24	1008.98 ± 162.62	410.89 ± 39.61		372.10 ± 25.82	391.52 ± 40.08		361.34 ± 27.10	388.88 ± 46.44		359.59 ± 21.40	792.58 ± 129.55		757.35 ± 93.49	870.87 ± 140.85		843.60 ± 151.44
LnRMSSD*	3.31 ± 0.69^#^	4.13 ± 0.58^#^	1.14 ± 0.33^**†**^		1.07 ± 0.23^**†**^	1.18 ± 0.38^**†**^		1.09 ± 0.28^**†**^	1.22 ± 0.31^**†**^		1.14 ± 0.36^**†**^	2.84 ± 0.62		3.25 ± 0.89	3.13 ± 0.62^#^		3.82 ± 0.69^#^
LnRMSSD:RR*	− 3.60 ± 0.58^#^	− 2.77 ± 0.44^#^	− 4.88 ± 0.26^**†**^		− 4.85 ± 0.21^**†**^	− 4.79 ± 0.31^**†**^		− 4.79 ± 0.30^**†**^	− 4.74 ± 0.24^**†**^		− 4.74 ± 0.36^**†**^	− 3.82 ± 0.53^#^		− 3.37 ± 0.78^#**†**^	− 3.62 ± 0.49^#^		− 2.91 ± 0.53^#^
LnLF	5.66 ± 1.47	7.47 ± 0.73	− 0.34 ± 0.71		0.05 ± 1.14	− 0.97 ± 0.99		− 0.22 ± 1.02	− 1.39 ± 0.82		− 0.76 ± 0.64	5.55 ± 1.25		6.91 ± 1.57	6.41 ± 1.07		7.45 ± 1.13
LnHF*	5.69 ± 1.37^#^	7.28 ± 1.10^#^	1.01 ± 0.71^**†**^		0.81 ± 0.62^**†**^	0.97 ± 0.90^**†**^		0.75 ± 0.82^**†**^	1.07 ± 0.81^**†**^		0.79 ± 1.20^**†**^	4.66 ± 1.11		5.67 ± 1.82	5.22 ± 1.15^#^		6.52 ± 1.51^#^
LFnu	49.86 ± 21.81	52.41 ± 24.06	24.52 ± 15.85		35.25 ± 19.86	15.72 ± 10.30		35.48 ± 26.67	10.15 ± 7.26		23.80 ± 19.43	69.83 ± 11.07		75.46 ± 11.53	74.17 ± 17.80		70.65 ± 11.67
HFnu	50.14 ± 21.81	47.59 ± 24.06	75.29 ± 15.78		64.54 ± 19.79	84.08 ± 10.32		64.43 ± 26.65	89.75 ± 7.26		76.00 ± 19.61	30.17 ± 11.07		24.54 ± 11.53	25.83 ± 17.80		29.35 ± 11.67
LnLF:RR^2^	− 8.16 ± 1.35	− 6.34 ± 0.68	− 12.37 ± 0.74		− 11.78 ± 1.03	− 12.90 ± 0.88		− 12.00 ± 0.91	− 13.30 ± 0.63		− 12.53 ± 0.55	− 7.78 ± 1.10		− 6.34 ± 1.41	− 7.10 ± 1.01		− 6.00 ± 0.83
LnHF:RR^2^*	− 8.13 ± 1.24^#^	− 6.53 ± 0.86^#^	− 11.02 ± 0.60^**†**^		− 11.03 ± 0.57^**†**^	− 10.97 ± 0.76^**†**^		− 11.03 ± 0.88^**†**^	− 10.85 ± 0.69^**†**^		− 10.98 ± 1.17^**†**^	− 8.67 ± 0.95^#^		− 7.58 ± 1.60^#**†**^	− 8.29 ± 0.93^#^		− 6.93 ± 1.22^#^
Resp Rate (bpm)	13.08 ± 3.04	10.98 ± 3.86	29.80 ± 5.16		28.79 ± 6.45	34.41 ± 5.82		31.33 ± 7.41	37.91 ± 4.83		34.99 ± 7.71	15.31 ± 3.60		14.78 ± 3.47	12.52 ± 4.01		10.68 ± 3.91

All values are presented in means ± SD. RR = R-R interval, LnRMSSD = natural logarithm of the root mean square of successive difference of R-R intervals, LnRMSSD:RR = natural logarithm of the root mean square of successive difference of R-R intervals divided by the average R-R interval, LnLF = natural logarithm of the low frequency power, LnHF = natural logarithm of the high frequency power, LFnu = normalised low frequency power of R-R intervals, HFnu = normalised high frequency power of R-R intervals. LnLF:RR^2^ = natural logarithm of the low frequency power divided by the average R-R interval squared, LnHF:RR^2^ = natural logarithm of the high frequency power divided by the average R-R interval squared * Significant group-time interaction effect (*P* < 0.05), ^#^ significant group difference between masters and young cyclists, ^†^ significant within-group difference to baseline value

Within-group comparisons to resting values for both the masters and young cyclists demonstrated significantly decreased LnRMSSD, LnHF, LnRMSSD:RR and LnHF:RR^2^ at all exercise timepoints (*P* < 0.001). For both groups, LnRMSSD and LnHF returned to resting values at 15–20 min of recovery (*P* > 0.05). However, for LnRMSSD:RR and LnHF:RR^2^, the masters cyclists returned to resting values at 15–20 min of recovery, whilst the younger cyclists did not return to resting values until 35–40 min of recovery (*P* > 0.05). No significant interaction effects were reported for all other time domain and power spectrum HRV parameters (*P* > 0.05). Significant effects for time across rest, exercise and recovery were reported for all HRV parameters (*P* < 0.001).

No significant group*time interaction effect was observed for respiratory rate (*P* > 0.05). Both the onset and offset LnRMSSD and LnRMSSD:RR data demonstrated no significant group*time interaction effects (*P* > 0.05) but significant time effects (*P* < 0.001).

## Discussion

The aim of this study was to compare HRV-derived parameters of cardiac autonomic regulation in well-trained masters and young cyclists during and following a high-intensity continuous cycle protocol. The present study found significant interaction effects for several HRV-derived indices of parasympathetic activity (LnRMSSD, LnHF, LnRMSSD:RR and LnHF:RR). Specifically, masters cyclists exhibited lower HRV parameters reflecting parasympathetic activity at rest and during recovery following a 60 min high-intensity continuous exercise bout compared to the younger cyclists, however, across the extended recovery period returned to the lower baselines values more rapidly than the younger cyclists. Given that the acute onset and offset data demonstrated no differences between the maters and young cyclists, this suggests that the acute exercise response and initial rates of recovery are similar between well-trained master and younger cyclists, but there may be an age effect on the absolute baseline values for HRV-derived parasympathetic metrics.

Although one previous study has compared the recovery of HRV-derived cardiac autonomic regulation following high-intensity interval training in masters and younger athletes (Borges et al. 2017), the present study is the first to compare the HRV response of masters and younger cyclists before (rest), during and following high-intensity continuous exercise. A novel finding of the present study was the lower resting values of parasympathetic activity in the masters athletes. Despite the fact that masters athletes have demonstrated higher levels of parasympathetic activity when compared to age-matched untrained counterparts (training effect) (Leti and Bricout 2013; Deus et al. 2019; Sotiriou et al. 2013; Galetta et al. 2005), few studies have compared masters athletes to training-matched younger counterparts (age effect). Previous studies comparing masters and younger athletes have reported either no differences in resting HRV indices (Borges et al. 2017) in masters athletes. Interestingly, the present study is the first to report lower parasympathetic activity at rest for masters athletes compared with younger athletes.

**Fig. 1:**
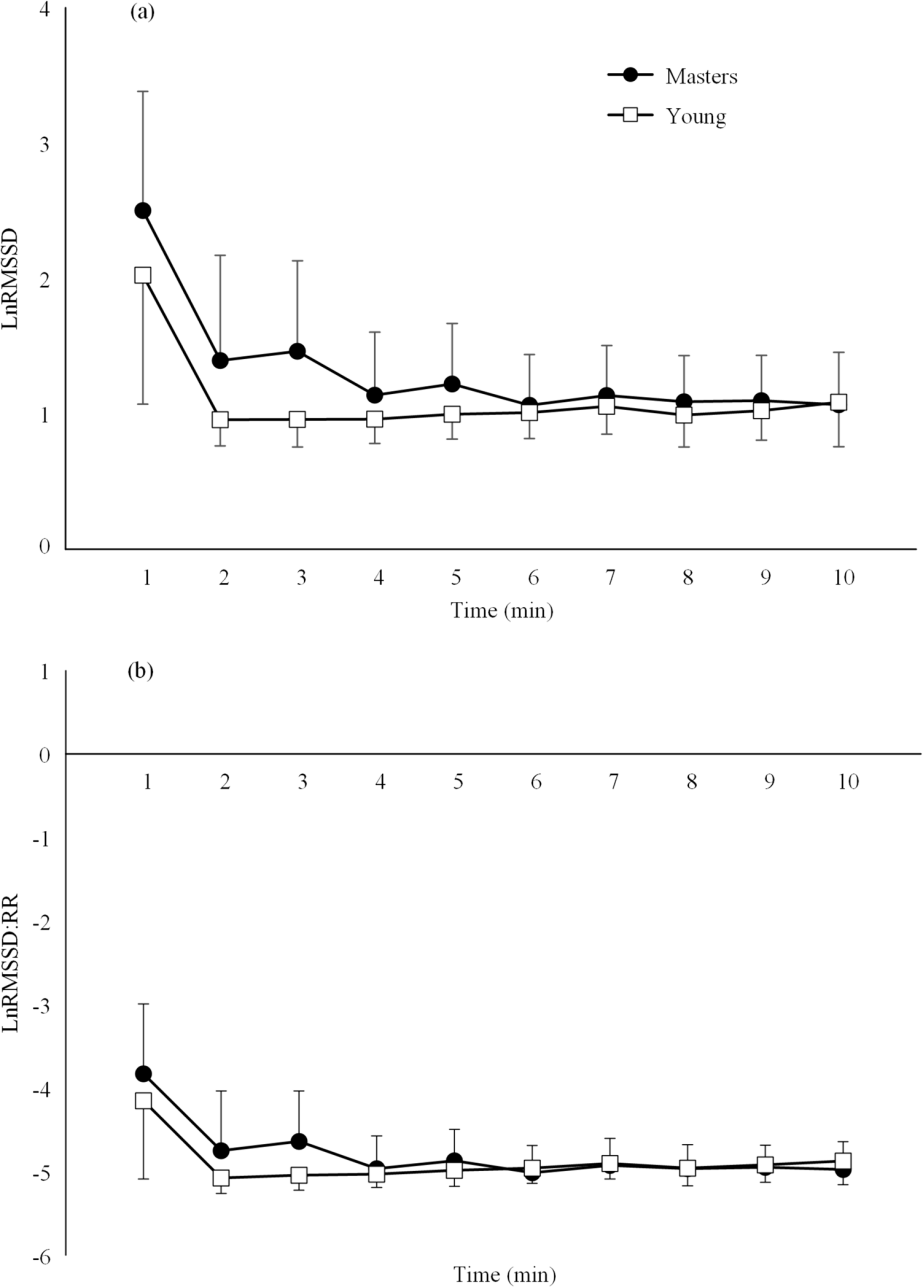
Mean ± standard deviation of the onset (**a**) root mean square of successive difference of the R-R intervals measured on successive 60 s segments (LnRMSSD) and the (**b**) R-R interval corrected RMSSD (LnRMSSD:RR) during the first 10 min of the continuous cycle protocol

Despite the significant differences in resting measures of HRV-derived parasympathetic activity, there were no significant age-related differences for any HRV parameter during exercise. Specifically, the acute onset period (Fig. 1) and the following 5-min segments (Table 2) during exercise demonstrated no between-group differences. In relation to the acute onset period, the authors decided to consistently apply 60-s non-overlapping segments to both onset and offset data for ease of comparison between onset of exercise and recovery following exercise. This method is common for offset data. However, previous studies investigating onset data have used smaller 15-s segments to provide a more sensitive analysis of the rapid onset response to exercise (D’Agosto et al. 2014; Nascimento et al. 2022; Oliver et al. 2023). As such, some of the onset response in the current study was likely lost due to the longer segment windows and may have masked any between-group differences during the onset period. Future research should explore the onset data in smaller segment windows to provide a more sensitive comparison of the rapid-onset response between master and younger athletes. (D’Agosto et al. 2014; Nascimento et al. 2022; Oliver et al. 2023) Nonetheless, the masters cyclists in this study demonstrated similar HRV responses to the younger cyclists during the CTS bout suggesting that the rate of vagal withdrawal and sympathetic activation during exercise were not impacted by age.

**Fig. 2:**
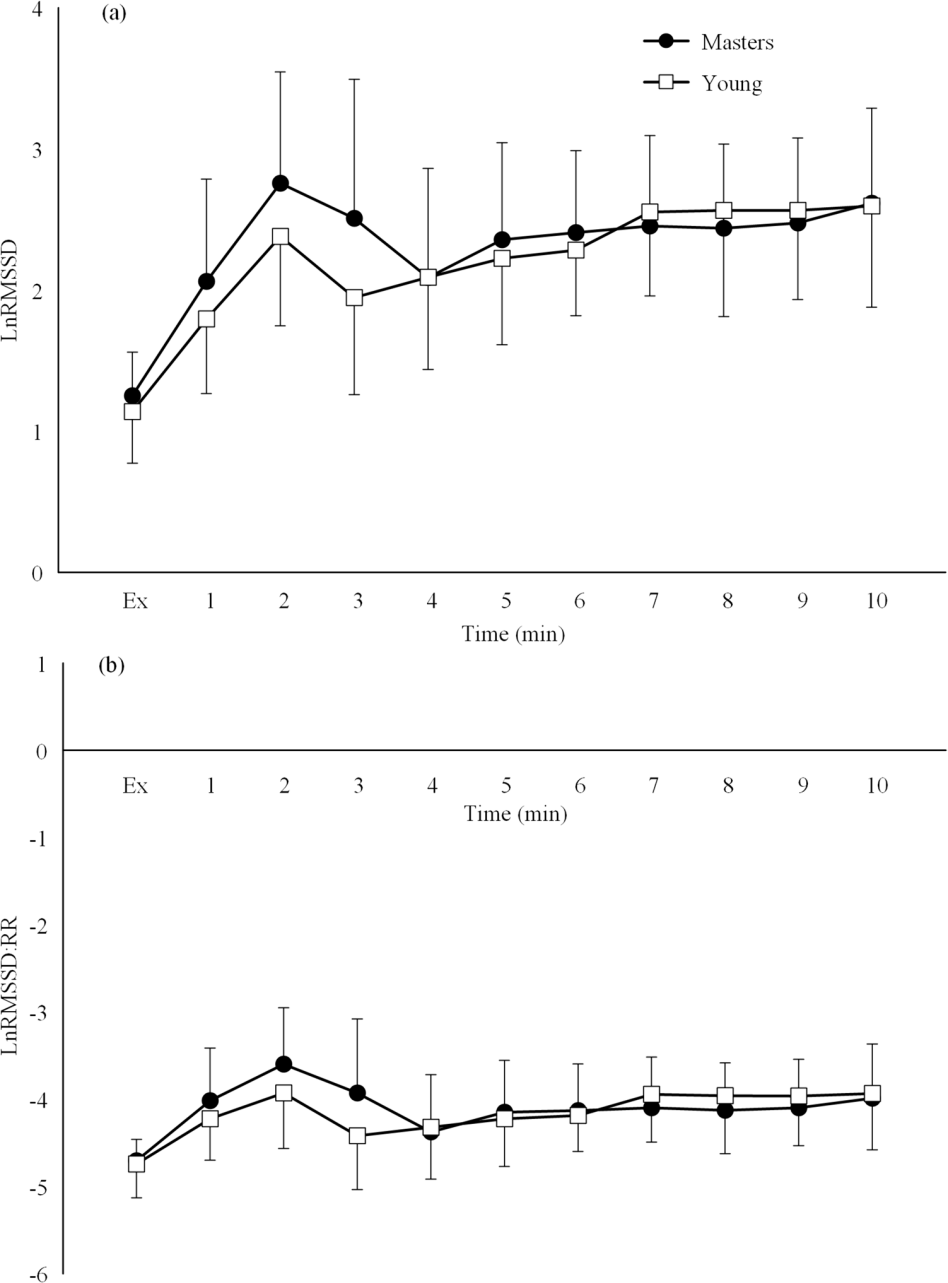
Mean ± standard deviation of the offset (**a**) root mean square of successive difference of the R-R intervals measured on successive 60 s segments (LnRMSSD) and the (**b**) R-R interval corrected RMSSD (LnRMSSD:RR) during the first 10 min of the recovery period

There were also similar between-group HRV responses during the 10-min offset period, immediately following the continuous endurance exercise cessation (Fig. 2). This is contrary to a previous finding that reported greater parasympathetic reactivation in masters cyclists compared to younger cyclists in the 10 min following a high-intensity interval protocol (Borges et al. 2017). These contrary findings are likely due to differences in the exercise protocol as both the intensity (Martinmäki and Rusko 2008) and duration (Michael et al. 2017c) of exercise have been demonstrated to influence the post-exercise HRV response. As such, the results from this study suggest that immediate parasympathetic reactivation following a CTS bout may not be impacted by age in athletic populations which may not be the case following high-intensity interval protocols. Despite this, following the initial 10-min offset period in the present study, age-related differences in HRV-derived parasympathetic activity become apparent further into the recovery period.

Similar to baseline values, the masters cyclists exhibited lower HRV-derived parasympathetic activation during recovery, particularly for heart rate corrected parameters (15–20 min and 35–40 min). However, given the lower baseline resting values for parasympathetic activity in masters cyclists, it is unsurprising the parasympathetic activity is lower in the extended recovery period as the athletes return to their baseline level. Indeed, within-group comparisons of the recovery response of LnRMSSD and LnHF relative to resting values demonstrate similar recovery trajectories for masters and younger cyclists as both groups returned to resting values by 15–20 min of recovery. Interestingly, the heart rate-corrected parameters suggest that the masters athletes actually returned to resting values by the 15–20 min of recovery, whereas the younger cyclists had only returned by the 35–40 min of recovery. It remains difficult to provide a conclusive statement about whether this suggests an accelerated parasympathetic reactivation in the masters athletes given the lower overall values for resting parasympathetic activity. It does, however, provide potential justification for removing the mathematical biases through correcting parameters to heart rate (R-R interval) (Billman 2013; Sacha 2013).

The authors acknowledge that a limitation of the present study is that blood pressure was not measured during exercise or recovery. Blood pressure changes have been shown to influence cardiac autonomic modulation (Carter et al. 2003) and may also be affected by age (Shantsila et al. 2015). Another potential limitation of the current investigation is that respiration rate was not controlled during recovery. However, the authors believe this is an unrealistic expectation during recovery in exercise trials. Although there were no significant differences between groups for respiration rate across the CTS and recovery protocol, the standard deviation range for the younger cyclists at rest and in the final period of recovery overlapped slightly with the lower bound of the spectral range. This suggests that some of the respiratory sinus arrhythmia for the younger cyclists may have been captured as low frequency and potentially confounded the frequency domain data at rest and the final recovery timepoint. However, given that the LnRMSSD (time domain) variables also demonstrated significant differences at the same timepoints as the LnHF variables, the authors feel that the HF activity and rapid heart rate modulation associated with respiratory sinus arrhythmia were adequately captured from a group level.

## Conclusion

This study provides novel insights into the effect age on HRV-derived parameters of cardiac autonomic regulation during and following a high-intensity continuous cycle exercise in master and young cyclists. The well-trained masters cyclists in the present study demonstrated similar exercise and immediate recovery responses in HRV variables, but demonstrated lower values for parasympathetic activity at rest and into extended recovery. It remains difficult to make conclusive comparisons on the rate of parasympathetic reactivation following exercise; however, HRV-derived cardiac autonomic regulation during continuous exercise and the immediate recovery period seem to be well-preserved through continued exercise training in masters endurance athletes, but extended recovery may be impacted by age.

## Data Availability

The data that support the findings of this study are available from the authors upon reasonable request.
